# Human immunodeficiency virus type 1 (HIV-1)-mediated neuroinflammation dysregulates neurogranin and induces synaptodendritic injury

**DOI:** 10.1186/s12974-018-1160-2

**Published:** 2018-04-27

**Authors:** Debjani Guha, Marc C. E. Wagner, Velpandi Ayyavoo

**Affiliations:** Department of Infectious Diseases and Microbiology, Graduate School of Public Health, University of Pittsburgh, 2117 Pitt Public Health, 130 DeSoto Street, Pittsburgh, PA 15261 USA

**Keywords:** HIV-1, Neurogranin, Calmodulin, Frontal cortex, Synaptodendritic damage

## Abstract

**Background:**

Human immunodeficiency virus type 1 (HIV-1)-associated neurocognitive disorder (HAND) is a common outcome of a majority of HIV-1-infected subjects and is associated with synaptodendritic damage. Neurogranin (Ng), a postsynaptic protein, and calmodulin (CaM) are two important players of synaptic integrity/functions. The biological role of Ng in the context of HAND is unknown.

**Methods:**

We compared the expression of Ng in frontal cortex (FC) tissues from control and HIV-1-positive subjects with and without HAND by immunohistochemistry, western blot, and qRT-PCR. The interaction between Ng and CaM was analyzed by co-immunoprecipitation. Ng, microtubule-associated protein 2 (MAP2), CaM, CaM-dependent protein kinase II (CaMKII), CREB, synaptophysin (Syp), and synapsin I (Syn I) expressions were evaluated by western blot using FC tissue lysates and differentiated SH-SY5Y (dSH-SY5Y) cells. Identification of inflammatory factors related to Ng loss was accomplished by exposing dSH-SY5Y cells to HIV-1 and mock-infected monocyte-derived macrophage (MDM) supernatants or HIV-1 NLYU2 pseudotyped with VSV-G-Env. Levels of interleukin (IL)-1β, IL-8, tumor necrosis factor (TNF)-α, monocyte chemoattractant protein (MCP)-1, MCP-2, and CXCL5 in MDM supernatants were measured by ELISA. Association of IL-1β and IL-8 to Ng expression in context of HIV-1 infection was evaluated in the presence or absence of neutralizing antibodies against these cytokines.

**Results:**

Expression level of Ng was reduced significantly in FC of HAND-positive (HAND+) patients compared to uninfected individuals. Although no difference was found in CaM expression, interaction between Ng and CaM was reduced in HAND+ patients, which was associated with decreased level of CaMKII, a downstream signaling molecule of CaM pathway. This in turn resulted in reduction of synaptic markers, Syp and Syn I. HIV-1 infection directly had no considerable effect on dysregulation of Ng expression in dSH-SY5Y cells, whereas high amount of pro-inflammatory IL-1β and IL-8 in HIV-1-infected MDM supernatants was associated with significant reduction in Ng expression.

**Conclusions:**

Synaptic damage in HAND+ patients could be a result of abrogation of Ng through HIV-1-induced inflammation that dysregulates Ng-CaM interaction and downstream signaling cascades associated with synaptodendritic functions. This is the first study evaluating the potential role of Ng in the context of HIV-1 neuropathogenesis.

## Background

Prolonged life expectancy of HIV-1-positive subjects in the post-combination antiretroviral therapy (cART) era has persisted the neurocognitive problems and currently higher number of people are living with HIV-associated neurocognitive disorder (HAND). About 30–50% of HIV-1-infected subjects suffer from mild to severe forms of neurocognitive abnormalities [1, 2]. Synaptic disruption in addition to neuronal death is one of the underlying mechanisms of neurocognitive impairment in HIV-1-infected patients. In contrast with HIV-encephalitis (HIVE) and neuronal apoptosis, synaptodendritic injury due to HIV-1 infection is more directly related to cognitive impairments among HAND patients [3]. Multiple proteins are involved in synaptodendritic function, and calmodulin is one of the important regulators for synaptic integrity and regulates phosphorylation and activation of different synaptic proteins. For instance, formation of Ca^2+^-CaM complex is followed by activation of various isoforms of Ca^2+^/CaM-dependent protein kinase II (CaMKII), high concentration of which has been reported to be associated with the increased strength of synaptic plasticity [4]. Hence, dysregulation of calmodulin (CaM) pathway is likely to be involved in synaptic damage induced by HIV-1 infection.

Postsynaptic protein neurogranin (Ng), mostly localized at the dendritic shafts and spines, is one of the regulatory factors for the availability of free CaM. Ng binds to Ca^2+^-free CaM to form Ng-CaM complex. However, the function of Ng-CaM complex is not well defined. Previous studies have shown that Ng could either sequester CaM in the vicinity of its downstream effectors particularly CaMKII and thereby inhibit the activation of the target molecules or it could concentrate and/or target CaM within dendritic spines to facilitate Ca^2+^/CaM-mediated signaling [5–8]. Studies have shown that Ng enhances calcium-mediated long-term potentiation (LTP) and transgenic mice lacking Ng results in impaired spatial learning tasks, and antibodies against Ng prevent LTP in hippocampal neurons in vitro [9, 10]. Although the pivotal role of Ng in a number of neurological diseases (Alzheimer’s disease, Parkinson’s disease, stroke) has been demonstrated [11–14], very little is known about the mechanistic role of Ng in the context of HIV-1 infection and HAND. Here, we evaluated for the first time the expression and functional role of Ng in HIV-1-infected individuals with and without HAND.

The inflammatory nature of infected macrophages/microglia in addition to HIV-1 viral proteins present in the central nervous system (CNS) are the major factors responsible for neuronal dysfunction [15–20]. Studies have demonstrated synaptic injury via exposure to viral proteins, Tat and gp120 [21, 22]. In addition, proinflammatory products released by HIV-1-infected macrophages have a role in synaptodendritic injury [3] supporting that HIV-1 impairs synaptic integrity. In this study, we delineated the mechanistic role of Ng in HIV-1-induced synaptic damage through CaM-CaMKII signaling pathway. Our results suggest that HIV-1 infection significantly downregulates the expression of Ng at the advanced stage of HAND, i.e., HIV-1-associated dementia (HAD). Furthermore, reduced Ng level results in loss of interaction with CaM leading to decreased expression of downstream protein CaMKII and synaptic markers Syp and Syn I. Additionally, proinflammatory cytokines, especially IL-1β and IL-8, caused significant reduction in Ng expression that could result in dysregulation of downstream CaM signaling molecules and disruption of the synapses. These observations, taken together, demonstrate that HIV-1-induced inflammation in the CNS results in synaptic damage through Ng dysregulation.

## Methods

### Brain tissues from HIV-1-positive and control subjects

Age- and sex-matched human frontal cortex (FC) tissues (frozen tissues and formalin fixed slides) from eight HIV-1-positive subjects with and without cognitive impairment and four HIV-1-negative subjects were obtained from *National NeuroAIDS Tissue Consortium* (NNTC) and multicenter AIDS cohort study (MACS) using appropriate IRB and CORID approval. Cognitive impairment included either HIV-1-associated dementia (HAD) or mild neurocognitive disorder (MND), and all of them were on cART. The demographic and clinical backgrounds of the study subjects are shown in Table 1.

**Table 1:** Demographic and clinical characteristics of study subjects

Subjects	HIV status	Age (years)	Gender	HAND diagnosis	Plasma viral load (copies/ml)	CD4 count (cells/μl)	ART
1	Negative	53	Male	–	–	–	–
2	Negative	51	Male	–	–	–	–
3	Negative	52	Male	–	–	–	–
4	Negative	62	Male	–	–	–	–
5	Positive	54	Male	Normal	113	18	Yes
6	Positive	67	Male	Normal	< 50	347	Yes
7	Positive	52	Male	Normal	82	317	Yes
8	Positive	66	Male	Normal	< 12	465	Yes
9	Positive	50	Male	MND	NA	24	Yes
10	Positive	56	Male	HAD	452,705	8	Yes
11	Positive	49	Male	HAD	246,267	13	Yes
12	Positive	43	Male	HAD	201,702	56	Yes

### Virus preparation and characterization

HIV-1 virus particles were generated using the pNL43-YU2-Env EGFP (CCR5-receptor utilizing strain) proviral DNA constructs. HEK293T cells in 10-cm plate were transfected with 5 μg of HIV-1 proviral or vector DNA using polyjet reagent (SignaGen Laboratories, MD, USA) as per manufacturer’s instructions. Supernatants were collected 72 h post-transfection, spun at 3000 rpm for 10 min, and filtered through a 0.4-μm filter to remove cellular debris. All virus stocks were further concentrated by ultracentrifugation at 22,000 rpm for 1 h at 4 °C and were assessed for infectivity using TZM-bl assay. For infecting/exposing neuronal (differentiated SH-SY5Y) cells, HIV-1 NL-YU2 virus particles were pseudotyped with vesicular stomatitis virus envelope (VSV-G-Env).

### Infection of MDMs

Monocytes were isolated from normal peripheral blood mononuclear cells (PBMC) and differentiated for 7 days. Briefly, CD14^+^ monocytes were purified from PBMC by positive selection using anti-CD14 monoclonal antibody-coated magnetic microbeads (Miltenyi Biotech) and differentiated in the presence of M-CSF and GM-CSF as described previously [23]. MDMs were infected with a multiplicity of infection (MOI) of 0.5. Mock infection was performed using equal amount of HEK293T supernatant. MDM supernatants were collected 10 to 12 days post-infection.

### Differentiation of SH-SY5Y cells and exposure

Neuroblastoma (SH-SY5Y) cells were cultured and differentiated into neuron as described by Dwane et al. (Dwane et al. BMC Research Notes 2013). In brief, SH-SY5Y cells were maintained in Dulbecco’s modified Eagle’s medium (DMEM) supplemented with 10% fetal bovine serum (FBS), 10 mM l-glutamine, and 1% penicillin/streptomycin medium. For differentiation, cells were cultured in DMEM containing 3% FBS and 10 μM *trans*-retinoic acid (RA). Medium was changed every other day for 7 days. Post-differentiation, SH-SY5Y cells were either infected with neurotropic HIV-1 NLYU2 virus at an MOI of 1.0 for 72 h or exposed to mock or HIV-1-infected MDM supernatants for 24 h. For exposure assays, MDMs were infected with R5 tropic HIV-1 NLYU2 at an MOI of 0.5 or mock for 10–12 days and supernatants from HIV-1 and mock-infected MDMs were used to expose differentiated SH-SY5Y cells (dSH-SY5Y) for 24 h. For neutralization assays, HIV-1-infected MDM supernatants were incubated for 2 h with neutralizing antibody against IL-1β (5 μg/ml) or IL-8 (5 μg/ml). dSH-SY5Y cells were exposed to HIV-1-infected MDM supernatants containing neutralizing antibody and harvested 24 h post-exposure.

### Subcellular fractionation

Separation and extraction of cytoplasmic and nuclear extracts from cells were performed using NE-PER nuclear and cytoplasmic extraction reagents according to manufacturer’s protocol (Thermo Fisher). Briefly, control and dSH-SY5Y cells exposed to MDM supernatants were treated with ice-cold CER-I and CER-II reagents to extract the cytoplasmic fraction, and the nuclear fraction was extracted by NER reagent provided in the kit.

### Measurement of cytokines by ELISA

Supernatants were collected from HIV-1- or mock-infected MDMs (*N* = 5) at 8 to 12 days post-infection and stored at − 80 °C. Concentrations of TNF-α, IL-1β, IL-8, MCP-1, MCP-2, and CXCL5 in the HIV-1 and mock-infected supernatants were measured by ELISA following the manufacturer’s protocol (R&D Systems, Minneapolis, MN, USA).

### Immunohistochemistry (IHC)

Human FC tissues along with formalin-fixed paraffin-sectioned slides either from control or HIV-1-positive individuals with or without HAND were obtained from NNTC using appropriate institutional IRB and CORID approval. For IHC, samples went through a process of heating at 60 °C for approximately 14 h. This was followed by deparaffinization involving several changes of xylene at 15 min each and succession of decreasing ethanol concentrations of 100, 95, and 70% then to distilled water. Slides were then subjected to heat-induced epitope retrieval (HIER) utilizing citrate buffer at pH 6.0 in an electric pressure cooker at high heat for 15 min followed by a cooling down period prior to the initiation of IHC protocol. Tissues were treated with hydrogen peroxide to block endogenous peroxidase activity as part of 3rd Generation IHC Detection Kit (Invitrogen, CA). This was followed by blocking with 10% normal goat serum for 15 min prior to incubation with primary antibody. Anti-Ng antibody (EMD Millipore) was utilized at a 1:1000 dilution, and the tissue sections were incubated at 4 °C overnight. The slides were developed utilizing the same 3rd Generation IHC Detection Kit, dehydrated, and mounted using permount.

### Immunofluorescence staining

Treated and untreated dSH-SY5Y cells were fixed in 4% paraformaldehyde for 15 min and permeabilized with 0.1% Triton-X-PBS for 15 min. The cells were rehydrated by three washes of PBS and five washes of 0.5% bovine serum albumin (BSA). After blocking with 2% BSA for 1 h, dSH-SY5Y cells were incubated with primary antibodies against Ng (1:200) and microtubule-associated protein 2 (MAP2) (1:250) overnight at 4 °C. Cells were washed five times with 0.5% BSA and were further incubated with Alexa Flour 488 goat anti-mouse-IgG and anti-rabbit-Cy3 followed by five washes with 0.5% BSA with PBS, and the nuclei were stained with Hoechst 33342 (1 μg/ml). The slides were mounted, and images were taken using confocal microscope.

### Coimmunoprecipitation (Co-IP)

Tissue lysates containing 100 μg equivalent of protein were first pre-cleared with isotype control followed by incubation with 1 μg of primary antibody at 4 °C for 60 min with gentle agitation. A/G plus agarose beads (20 μl) were added and incubated for overnight at 4 °C. The mixture was centrifuged at 3000 rpm for 5 min, and the supernatant was discarded. Antibody-bound protein complex was washed, and the pellet was suspended in a sample buffer for western blot.

### Western blot

Differentiated SH-SY5Y cells (infected or exposed to MDM supernatants) were washed twice with cold PBS and lysed in buffer containing 50 mM Tris (pH 7.5), 150 mM NaCl, 1% Triton X-100, 1 mM sodium orthovanadate, 10 mM sodium fluoride, 1 mM phenylmethyl-sulfonylfluoride, 0.05% deoxycholate, 10% SDS, 0.07 trypsin inhibitor units/ml aprotinin, and protease inhibitors leupeptin, chymostatin, and pepstatin (1 μg/ml). About 5 mg of frozen human FC tissues were cut into small pieces and homogenized in ice-old RIPA buffer containing 1 mM PMSF. Tissues were agitated in lysis buffer for 2 h at 4 °C. Cells and tissue lysates were clarified by centrifugation at 10,000 rpm for 15 min, and the total lysates (30 μg protein equivalent for cells and 5 μg for tissues) were separated on a SDS-PAGE gel and transferred, and protein expression was detected with anti-Ng (EMD Millipore) (1:5000), anti-CaM (1:10000), anti-CREB (1:1000), anti-CaMKII (1:1000), anti-synaptophysin (1:5000), and anti-BDNF (1:1000). Tubulin (Cell Signaling Technology) was used as the loading control. Blots were developed using the ECL kit (Pierce). Band intensity was quantitated by the ImageJ software, and densitometry measurements were normalized against MAP2 or tubulin.

### Real-time PCR

Total RNA was extracted from HIV-1-positive and HIV-1-negative human FC tissues as well as from control, and treated dSH-SY5Y cells using the RNeasy mini kit according to the manufacturer’s protocol (Qiagen, Valencia CA, USA). Quantitative real-time PCR was performed to determine the expression of Ng following manufacturer’s protocol (Life Technologies) and normalized with MAP2 (neuronal marker) as control. Fold difference was calculated by −∆∆Ct method.

### Statistical analysis

In vivo data were analyzed by nonparametric Mann-Whitney test for non-normally distributed data using the GraphPad Prism software. In vitro data were analyzed by Student’s *t* test. Results were expressed as mean ± SEM for at least three experiments, and *p* < 0.05 was considered as significant. IHC pictures were analyzed using NIS Elements, and western blot band intensities were measured by the ImageJ software.

## Results

### Effect of HIV-1 infection and/or HAND on Ng expression

**Fig. 1:**
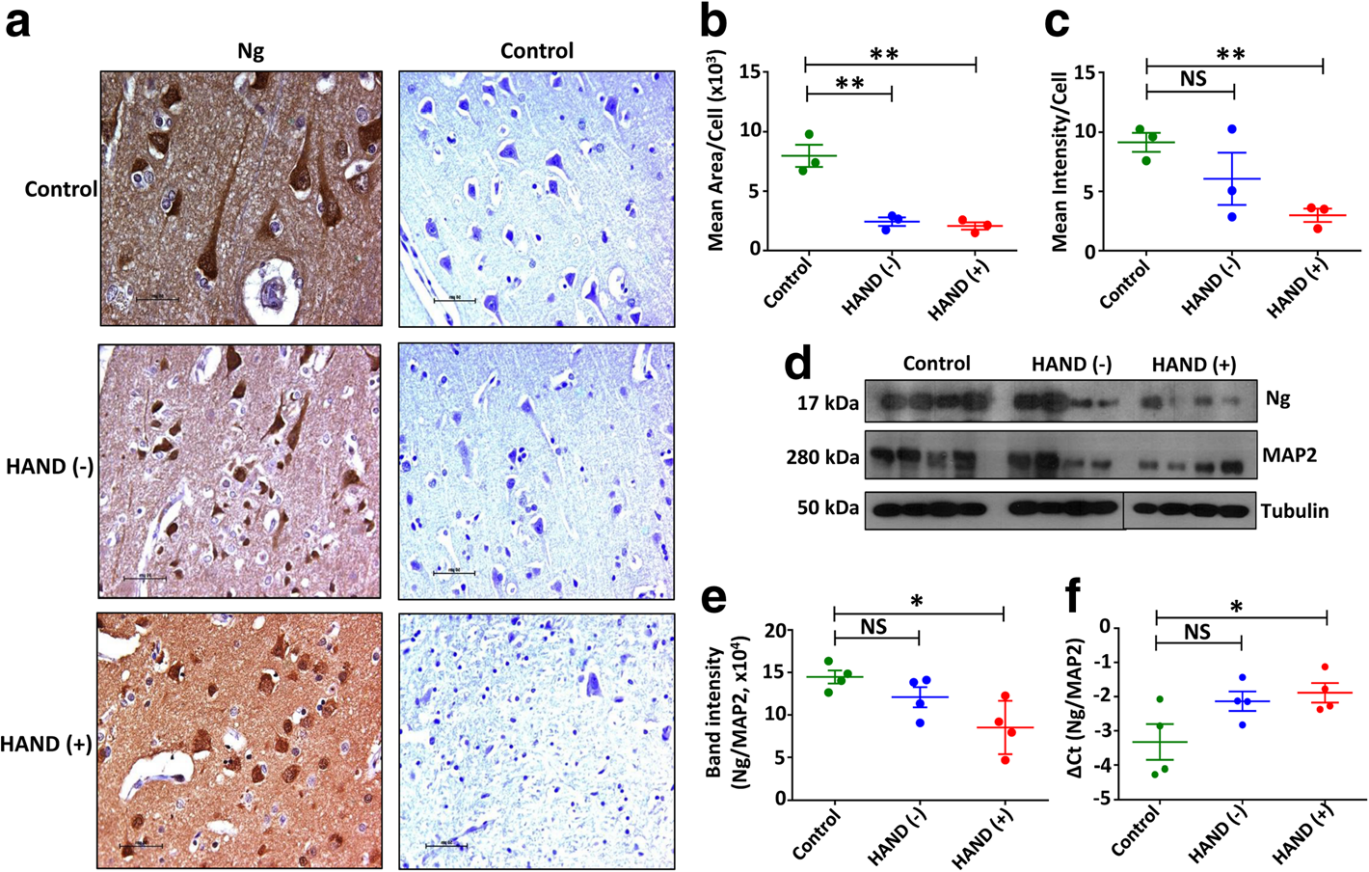
HIV-1 infection and HAND pathology dysregulate Ng expression in human FC tissue: **a** FC sections from uninfected control, HAND (−), and HAND (+) subjects were IHC-stained for Ng and counterstained with hematoxylin. Scale bar indicates 50 μm. **b**, **c** The area and intensity of Ng-positive cells were counted from five different fields from each subject and divided by the total number of cells in those fields to calculate the mean area and mean intensity per cell. Positive cells were masked based on signal intensity threshold and normalized over noise using the NIS Elements software. Selection of an appropriate background and shading correction as well as smoothing filter object minimized noise of the images, allowing for more accurate analyses. **d** FC tissues from control, HAND (−), and HAND (+) subjects were homogenized and lysed, and 5 μg of each lysate were loaded into each lane to measure Ng expression by western blot using specific antibody (*N* = 4 for each group). MAP2 was used as control for normalization. Tubulin was used as a loading control. **e** Band intensities were quantitated by the ImageJ software, and the data were normalized with MAP2 and tubulin. **f** qRT-PCR was performed with Ng-specific primers and probes using the RNA isolated from the frozen tissues from the same subjects (*N* = 4 for each group). Results were normalized with housekeeping gene RPLPO. Results are the ±SEM of four individual experiments, nonparametric Mann-Whitney tests were performed to calculate significance, and *p< 0.05 and **p < 0.01 compared to control. NS not significant

Earlier studies have implicated a role for Ng in brain diseases, such as Alzheimer’s disease, Parkinson’s disease, schizophrenia, epilepsy, and other neurodegenerative diseases; however, there is lack of understanding of the function of Ng in the context of HIV-1 infection or HAND. To determine whether Ng has any functional role in HAND pathogenesis, we analyzed FC tissues from eight HIV-1-infected subjects with and without cognitive impairment and four HIV-1-negative control subjects. Ng expression by IHC showed marked reduction of Ng level in HAND (+) subjects, compared to the control subjects. The major changes observed in HIV-1-positive FC tissues were the loss of dendrites as well as increased granularity of Ng (Fig. 1a). Quantitation of the expression of Ng in all three groups was determined by comparing mean area/cell and the mean intensity/cell. The mean area/cell was reduced significantly in both HIV-1-positive HAND (−) (*p* = 0.005) and HAND (+) (*p* = 0.004) patients compared to the uninfected control group (Fig. 1b). Mean intensity was significantly lower in HAND (+) patients (*p* = 0.003); however, no significant difference was found in HAND (−) subjects compared to control subjects (Fig. 1c). IHC results were further confirmed by western blot using FC tissue lysates. The results were normalized with respect to another neuronal marker, MAP2 expression (Fig. 1d), which was normalized to tubulin. HAND (+) subjects exhibited reduced expression of Ng in the FC compared to uninfected control group (*p* = 0.03) (Fig. 1e). HIV-1-positive and cognitively normal subjects also showed similar trend of reduced Ng level compared to controls. Similar difference in control, HAND (−), and HAND (+) groups was also observed at the Ng RNA level (Fig. 1f) suggesting that HIV-1-induced downregulation of Ng expression might be regulated both at the RNA and protein level.

### HIV-1-induced reduction of Ng expression dysregulates CaM-CaMKII pathway

**Fig. 2:**
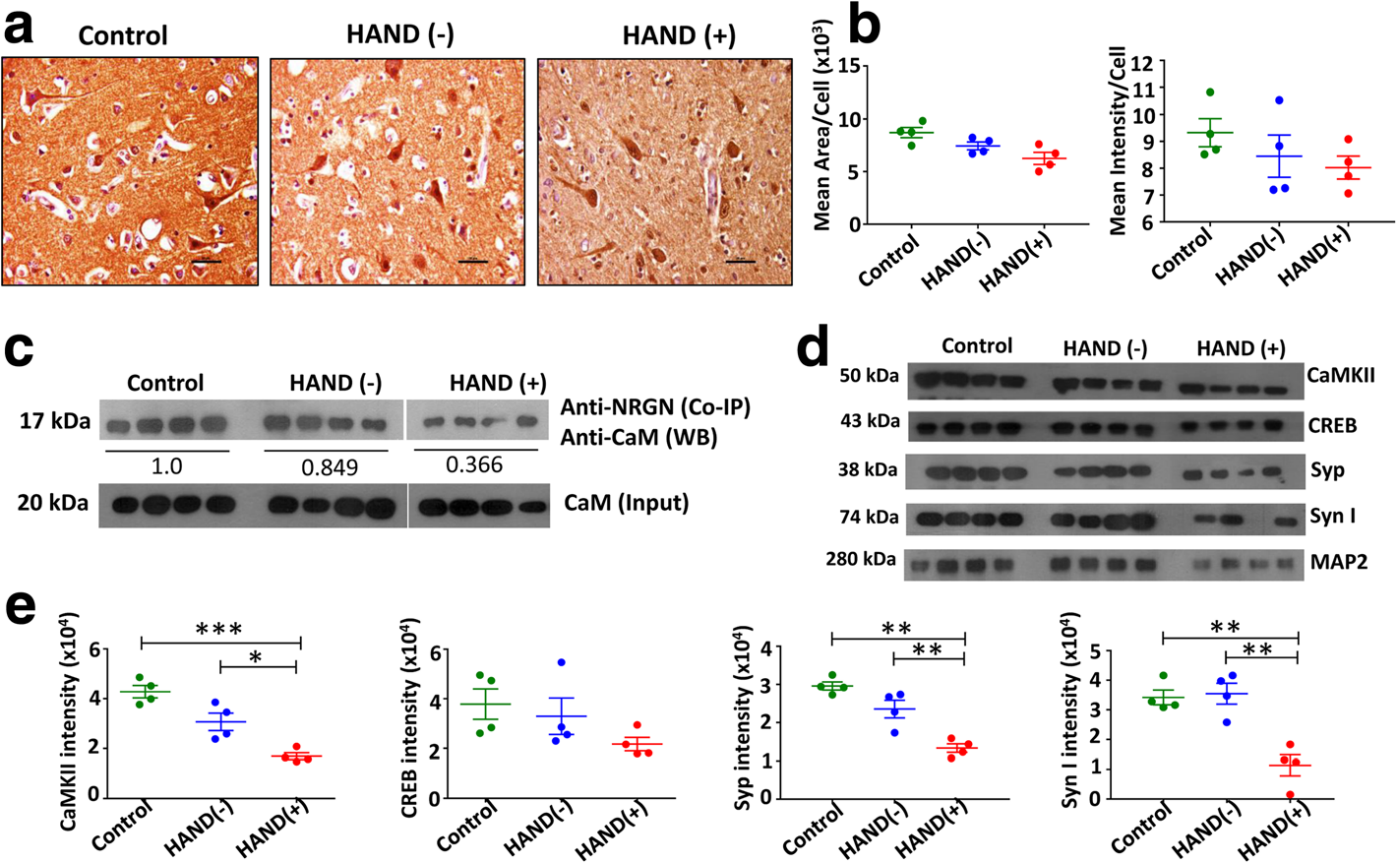
Decreased Ng-CaM interaction reduces the expressions of synaptic plasticity markers in HAND patients. **a** IHC staining of CaM in the FC tissues from control, HAND (−), and HAND (+) subjects were performed. Scale bar indicates 50 μm. **b** Mean area/cell and mean intensity/cell were calculated from five different fields from each subject using the NIS Elements software. **c** Lysates prepared from control, HAND (−), and HAND (+) FC tissues were subjected to co-IP using anti-Ng antibody. Western blot was performed using anti-CaM antibody. Each lane represents sample from a single donor. Input represents presence of CaM in samples used for co-IP. **d** Control, HAND (−), and HAND (+) FC tissues were lysed, and 5 μg of each lysate was analyzed by western blot using antibodies against CaMKII, CREB, Syp, Syn I, and MAP2. **e** Relative band intensities were normalized with MAP2. Densitometry quantification of western blot data represents ± SEM of four independent observations. *p < 0.05 compared to control. NS not significant

Ng is known to interact with CaM and regulate the availability of CaM in the dendritic spines to induce synaptic plasticity [7, 8]. Hence, to determine whether HIV-1 infection alters CaM level, we performed IHC on the uninfected control, HAND (−), and HAND (+) FC tissues. No significant difference in CaM expression was obtained in any of these groups (Fig. 2a, b). Next, we investigated by co-IP whether HIV-1-induced reduction of Ng concentration could result in alteration of Ng-CaM interaction in HAND (−) or HAND (+) subjects. Briefly, FC tissue lysates from all three groups with equivalent amount of CaM were lysed and immunoprecipitated with anti-Ng antibody to pull down Ng-CaM complex followed by western blot using anti-CaM antibody. The average band intensity was compared among the groups and normalized against the control group. As presented in Fig. 2c, HAND (+) subjects exhibited significantly reduced interaction of Ng with CaM in comparison to the control group (0.366-fold compared to control, *p* < 0.05). Since CaMKII and CREB are known to strengthen synapses, we assessed whether reduced Ng-CaM interaction in HAND (+) subjects results in alteration of the downstream molecules of the CaM-CaMKII pathway. Tissue lysates from the three groups were used to perform western blot using anti-CaMKII and anti-CREB antibodies (Fig. 2d). MAP2 band intensities were first normalized with tubulin, and the intensities of CAMKII and CREB bands were normalized with MAP2. CaMKII expression was reduced by an average of 2.5-fold (*p* = 0.0004) compared to control subjects and 1.8-fold (*p* < 0.05) compared to HAND (−) subjects. A similar trend of reduction of CREB was also observed in HAND (+) subjects compared to uninfected control subjects (Fig. 2e).

### HIV-1 infection induces synaptic damage in HAND-positive subjects

Evaluation of synaptic dysfunction by measuring LTP with postmortem brain tissues was beyond our scope; hence, we selected two proteins, Syp and Syn I, that have potential roles in synaptic function such as synapse formation, biogenesis, synaptic transmission, and synaptic plasticity [24–26]. To assess the effect of HIV-1 infection on synaptic integrity and/or function, Syp and Syn I were analyzed by western blot in FC tissue samples along with CaMKII and CREB (Fig. 2d). Levels of both these proteins were decreased significantly in HAND (+) subjects compared to control (*p* = 0.002 for Syp and *p* < 0.0001 for Syn I) and HAND (−) subjects (*p* = 0.007 for Syp and *p* = 0.003 for Syn I) (Fig. 2e). No difference was found between HAND (−) and control subjects. Thus, these results suggest that HIV-1 infection and HAND pathogenesis alter the synaptic proteins through Ng dysregulation indicating synaptic dysfunction.

### HIV-1 infection indirectly dysregulates Ng expression in vitro

**Fig. 3:**
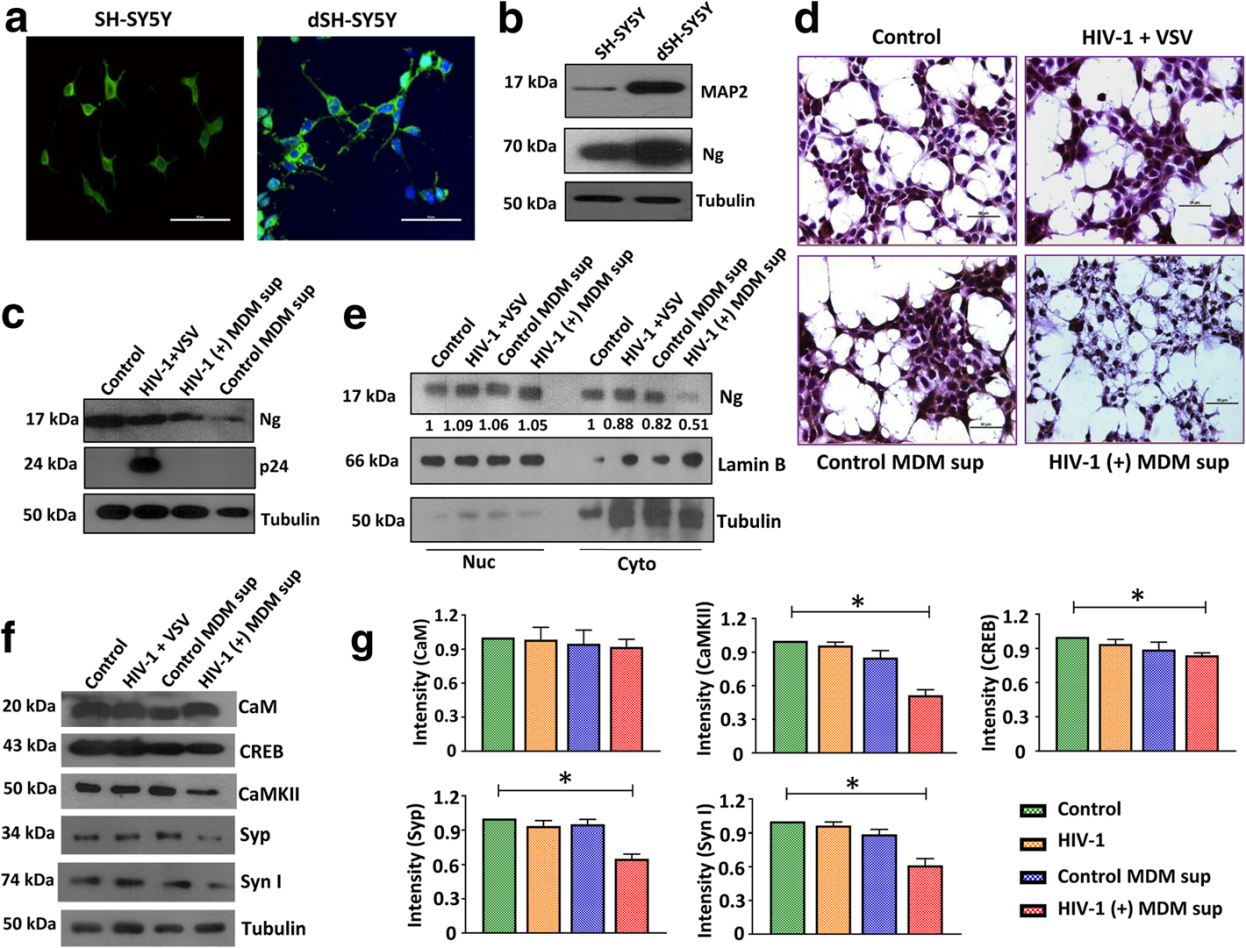
Delineating the host cellular factors responsible for Ng deregulation. **a** Neuroblastoma cell line, SH-SY5Y cells were differentiated with RA and immunostained with MAP2-specific antibody. Green represents MAP2, and blue represents DAPI (nucleus). Ng expression increases post-differentiation of SH-SY5Y cells. Scale bar indicates 50 μm. **b** Differentiated (dSH-SY5Y) and undifferentiated SH-SY5Y cells were lysed and 30 μg of protein was loaded, and expression of MAP2 and Ng was measured by western blot. Tubulin was used as a loading control. **c** dSH-SY5Y cells were either infected with HIV-1 NLYU2 pseudotyped with VSV-G at an MOI of 1.0 for 72 h or exposed to HIV-1 or mock-infected MDM supernatants for 24 h. Expression of Ng in transduced or exposed cells was analyzed by western blot (*N* = 3). **d** Control, transduced, or exposed dSH-SY5Y cells were stained for Ng and counterstained with hematoxylin. Scale bar represents 50 μm. **e** Cytoplasmic and nuclear fractions of the control, transduced, or exposed dSH-SY5Y cells were examined for the presence of Ng by western blot. Lamin B and tubulin were used as nuclear and cytoplasmic markers. Nuclear and cytoplasmic Ng band intensities were normalized over lamin B and tubulin, respectively. **f** Cell lysates from control, transduced, or exposed dSH-SY5Y cells (30 μg) were examined for the expression of CaM, CaMKII, CREB, Syp, and Syn I by western blot. Tubulin was used as a loading control. **g** Relative band intensities were normalized with tubulin. Densitometry quantification of western blot data represents ±SEM of three independent observations. *p < 0.05 compared to control

Results described above indicate that HIV-1-positive subjects with HAND exhibit distinct Ng expression and related alterations of the downstream molecules associated with Ng pathway in vivo. Next, we sought to determine the factors responsible for the changes in Ng expression in the neurons. For this purpose, we used differentiated SH-SY5Y (dSH-SY5Y) cells. The neuronal phenotype of the differentiated cells was first confirmed by immunostaining (Fig. 3a) and western blot (Fig. 3b) using anti-MAP2 antibody. MAP2 positivity by immunofluorescence staining indicates 80% differentiation of SH-SY5Y cells. Interestingly, differentiated cells exhibited an increase in Ng expression compared to the undifferentiated cells. Hence, all the in vitro experiments were conducted using the dSH-SY5Ycells.

To further identify whether viral factors have a role in altering Ng expression, dSH-SY5Y cells were infected with either neurotropic HIV-1 NLYU2 virus pseudotyped with VSV-G at an MOI of 1 or mock for 72 h. To assess the role of HIV-1-induced inflammatory factors in regulating Ng expression, MDMs were infected with R5 tropic HIV-1 NLYU2 at an MOI of 0.5 or mock for 10–12 days (*N* = 5). dSH-SY5Y cells were exposed to HIV-1 or mock-infected MDM supernatants for 24 h, and the effect of HIV-1 infection and/or inflammatory factors on Ng expression was analyzed by western blot (Fig. 3c) and immunocytochemical staining (Fig. 3d). As expected, virus infection alone did not significantly affect Ng expression, whereas exposure to HIV-1-infected MDM supernatants decreased Ng level drastically in dSH-SY5Y cells compared to mock-infected MDM supernatant-exposed cells. Supernatants from mock-infected MDMs reduced the level of Ng expression by 1.8-fold, whereas supernatants from HIV-1-infected MDMs reduced the Ng expression by 4.7-fold (*p* < 0.05). Analyses of immunostaining data showed that treatment of dSH-SY5Y cells with HIV-1-infected MDM supernatants resulted in loss of Ng specifically from dendrites in addition to abrogation of the architecture of the cells. Together, these results suggest that excessive inflammation in HAND (+) subjects could induce loss of Ng. This could be either specific loss of Ng from dendrites or translocation of the same from dendrites to nucleus. To examine this, subcellular fractionation was performed on control, HIV-1-infected, and MDM supernatant-exposed dSH-SY5Y cells and Ng expression was measured in both nuclear and cytoplasmic fractions. Lamin B and tubulin were used as markers for nuclear and cytoplasmic fractions, respectively. Nuclear and cytoplasmic Ng band intensities were normalized over lamin B and tubulin, respectively, and corrected for low level of contamination of other fractions. Following exposure of dSH-SY5Y cells to supernatants from HIV-1-infected MDMs, no change in Ng in the nuclear fraction was observed, whereas there was about 50% reduction of Ng expression in the cytoplasmic fraction (Fig. 3e). This result indicates that the loss of Ng occurs primarily in the cytoplasm and/or dendrites of dSH-SY5Y cells.

Next, to investigate whether treatment of dSH-SY5Y cells with HIV-1 infected MDM supernatants could alter CaM-CaMKII pathway, expressions of CaM, CaMKII, and CREB were examined by western blot. No significant change was observed in CaM and CREB expression in dSH-SY5Y cells exposed to HIV-1-infected MDM supernatant, whereas there was a significant (*p* = 0.03) reduction of CaMKII expression in exposed cells compared to the untreated control cells (Fig. 3f, g). Additionally, we also examined whether expression of the synaptic plasticity markers, Syp and Syn I, in dSH-SY5Y cells were altered post-exposure to the MDM supernatant or virus particles. Interestingly, it was noted that both Syp and Syn I expressions were reduced in dSH-SY5Y cells treated with HIV-1-infected MDM supernatant by 4.5- and 4.8-fold, respectively, compared to that in control dSH-SY5Y cells (Fig. 3f, g). This suggests that inflammation due to HIV-1 infection could have a potential role in decreasing Ng expression and causing synaptic damage.

### Cellular factors responsible for HIV-1-induced dysregulation of Ng expression

**Fig. 4:**
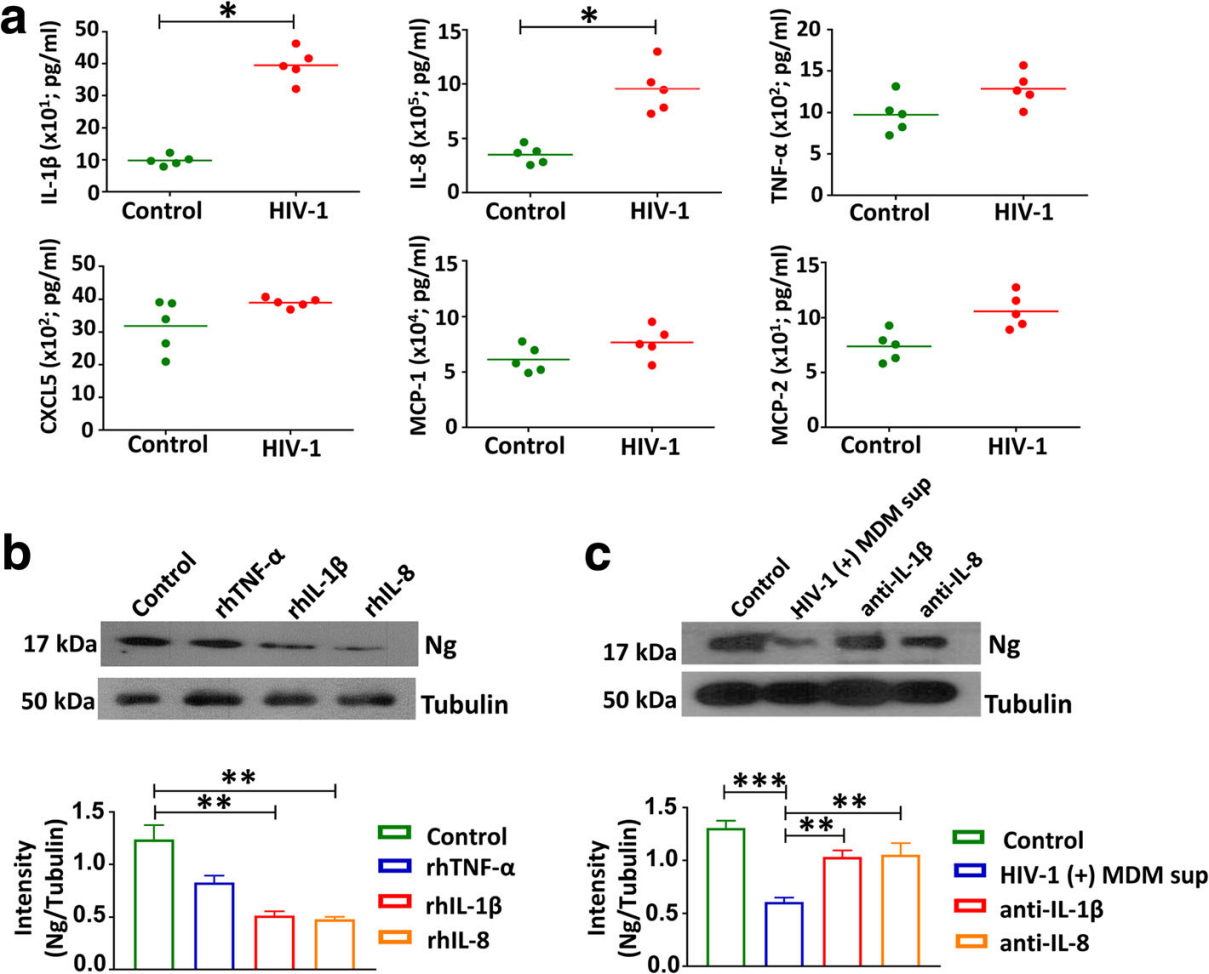
Role of proinflammatory cytokines in Ng dysregulation: Normal donor-derived MDMs were infected with HIV-1 NLYU2 at an MOI of 0.5 or mock for 10–12 days. **a** Levels of IL-1β, IL-8, TNF-α, CXCL5, MCP-1, and MCP-2 in supernatants of HIV-1 and mock-infected MDMs were measured by ELISA (*N* = 5). HIV-1 infection increased the production of proinflammatory cytokines. The horizontal lines represent mean concentrations of the cytokines. *p < 0.05 compared to mock-infected cultures. **b** dSH-SY5Y cells were treated with rhIL-1β (10 ng/ml), rhIL-8 (10 ng/ml), and rhTNF-α (100 ng/ml) for 24 h, and 30 μg of cell lysates for each sample was analyzed by western blot for Ng. Tubulin was used as a loading control. Band intensities from three independent experiments were measured by the ImageJ software. **c** dSH-SY5Y cells were exposed to HIV-1-infected MDM supernatants with or without anti-IL-1β or anti-IL-8 antibodies and harvested after 24 h of exposure. Cell lysates (30 μg) were analyzed by western blot for Ng. Band intensities from three independent experiments were measured by the ImageJ software. Densitometry quantification of western blot data represents ±SEM of three independent observations. *p < 0.05 and **p < 0.01 compared to control

Both HIV-1 viral proteins and inflammatory cytokines have deleterious effect on neurons. However, studies have shown that viral proteins also trigger inflammation and upregulate inflammatory cytokines [22]. Hence, we focused on identifying the proinflammatory cytokines/chemokines present in the HIV-1-infected MDM supernatants responsible for the alteration of Ng expression. For this purpose, we quantitated by ELISA the levels of cytokines/chemokines that are known to have roles in neuropathogenesis [23, 27–29]. Among the cytokines tested, we observed a significant increase in IL-1β (*p* = 0.0005) and IL-8 (*p* = 0.0012) in HIV-1-infected MDM supernatants compared to mock-infected supernatants. No significant difference was found in the level of TNF-α, MCP-1, MCP-2, and CXCL5 in those two groups (Fig. 4a).

To confirm that HIV-1-induced decreased expression of Ng is mediated at least in part through the proinflammatory cytokines released by infected MDMs, dSH-SY5Y cells were exposed to recombinant human IL-1β and IL-8 for 48 h. TNF-α was used as a control. Treatment of dSH-SY5Y cells with rhIL-1β and rhIL-8 exhibited 4.1-fold (*p* = 0.003) and 6.3-fold (*p* = 0.002) reduction of Ng expression, respectively, (Fig. 4b), whereas no significant reduction in Ng expression was observed with TNF-α treatment. To further confirm the role of IL-1β and IL-8 in regulation of Ng expression, HIV-1-infected MDM supernatants were incubated with IL-1β and IL-8 neutralizing antibodies or isotype controls for 24 h. dSH-SY5Y cells were exposed to the neutralizing antibody-treated MDM supernatants and Ng expression was determined by western blot. Exposure of dSH-SY5Y cells to HIV-1-infected MDM supernatants with anti-IL-1β and anti-IL-8 antibodies restored 69% and 73% Ng expression, respectively (*N* = 3) (Fig. 4c). Collectively, these results suggest that HIV-1-induced inflammation, specifically proinflammatory cytokines IL-1β and IL-8, have a potential role in Ng downregulation, which is associated with dysregulation of CaM-CaMKII pathway and synaptic damage.

## Discussion

Studies have established the role for Ng in CNS pathology, including Alzheimer’s disease, Parkinson’s disease, traumatic brain injury, and schizophrenia [30–34]. Ng functions as a potential biomarker for Alzheimer’s disease as higher level of Ng is consistently detected in the cerebrospinal fluid (CSF) of these patients [35, 36]. Higher level of Ng in CSF is a correlate of cognitive decline in neurodegenerative diseases. However, not much is known regarding the role of HIV-1 infection on Ng expression or the correlate of Ng-HAND. Thus, to understand the significance of Ng in HIV-1-positive subjects with or without HAND, we used human FC tissues from HIV-1-positive and HIV-1-negative subjects. Most of the HAND (+) subjects were at the advanced stage of cognitive impairment (HAD). Although HAD accounts for only 3–7% of HIV-1 positive cases in the post-cART era, we included mostly HAD subjects to understand the ultimate changes in Ng expression in HIV-1-infected patients. Our goal was to determine the changes in Ng expression and associated downstream molecules at the advanced stage of the disease in HIV-1-positive subjects. We have shown that advanced pathology of HAND abrogates Ng expression in the FC tissues. In HAND (+) subjects, the pattern of Ng expression in FC tissues is very distinct compared to the normal individuals. Both distribution/localization and intensity of Ng are significantly reduced, and more importantly, extensive granularity of Ng is observed in HAND (+) subjects. To assess whether the decrease of Ng expression is a secondary effect of neuronal degeneration, we used a second neuronal marker, MAP2, which is present in the neuronal cell bodies and dendrites of both the brain and spinal cord [37]. Results upon normalization of Ng with MAP2 indicate that the effect of HIV-1 on Ng was independent of MAP2 modification, suggesting that dysregulation of Ng could modulate the downstream synaptic functions of neurons and it may not be a correlative effect.

The major function of Ng is to control the intracellular concentration of CaM, which is essential for maintenance and function of synapses. In a recent study, it has been shown that increased Ng concentration enhances levels of CaM in the dendritic spines [38]. Thus, the level of Ng is one of the determining factors for CaM availability in the dendritic spines. Since the expression of Ng was reduced in HAND (+) subjects, we first sought to measure the concentration of CaM in uninfected, HIV-1-positive HAND (−), and HIV-1-positive HAND (+) groups. Surprisingly, following normalization with MAP2, no significant difference in the expression of CaM in any of the groups was noticed though reduction in dendritic length was observed in HAND (+) patients. This could be due to the fact that the cellular concentration of CaM is very low and the availability could be regulated by a number of other calpacitin proteins in addition to Ng. IHC or western blot may not be a very sensitive method to detect the small changes in CaM expression in FC tissues. Also, the expression of CaM is not similar in all regions of FC tissues; thus, screening more tissue sections of large number of FC samples may exemplify the significant reduction of CaM in HAND patients. However, due to the low availability of Ng in HAND (+) patients, there was a reduced Ng-CaM interaction (the interaction between Ng and CaM could be direct or indirect), which could potentiate synaptic damage. With frozen postmortem human brain tissues, it was not possible to examine the inhibition of LTP using electrophysiological assays. Therefore, we assessed the expression level of synaptodendritic injury markers Syp and Syn I along with CaMKII and CREB. Results indicate that limited interaction of Ng with CaM resulted in downregulation of CaMKII molecule involved in the CaM-CaMKII pathway. CaMKII is implicated in synaptic plasticity, and activation of this molecule could invoke various nerve functions including neurotransmitter synthesis, release, ion channel and receptor function, learning, and memory [39]. It has also been reported that Ng-mediated activation of CaMKII insert α-amino-3-hydroxy-5-methyl-4-isoxazolepropionic acid receptors (AMPARs) at the synapses, which contributes to the induction of LTP [40]. Hence, downregulation of CaMKII could lead to failure in recruitment of AMPAR, impairment of synaptic plasticity, and other nerve functions. Downregulation of CaMKII in turn loses its potential to activate CREB, one of the downstream molecules of CaMKII pathway. Activation of CREB is important to induce multiple gene expression related to spatial memory formation [41] including brain-derived neurotrophic factor (BDNF), Syp, and Syn I. The latter two are synaptic vesicle-associated proteins involved in formation and maintenance of synapses [25, 42]. With the reduction in Ng-CaM interaction, expressions of synaptodendritic markers were also reduced indicating synaptodendritic injury. Collectively, this suggests that Ng-CaM interaction is one of the regulating factors for synaptodendritic integrity. These results correlate with a previous study, which reported that Ng mutants unable to bind to CaM do not potentiate synaptic transmission and knockdown of Ng blocks induction of LTP [43]. Similar results of reduced level of all the synaptodendritic markers were also found in our in vitro study with dSH-SY5Y cells exposed to HIV-1-infected MDM supernatants. However, it is also important to point out that differentiated SH-SY5Y cells do not form mature synapses, though they are morphologically similar to primary neurons and express neuron-specific markers including CaMKII, CREB, Syp, and Syn I.

Activated monocytes/macrophages are involved in trafficking the infectious HIV-1 through blood-brain barrier (BBB) into the brain compartment initiating the infection in the CNS. These cells release HIV-1 viral proteins and inflammatory molecules in the CNS and thus function as vital players for neuronal damage [44, 45]. HIV-1-infected macrophages/microglia have been shown to cause neuronal calcium dysregulation and neurotoxicity, the effects of which can be inhibited by blocking extracellular calcium signaling [46, 47]. Thus, monocytes/macrophages are involved in maintenance as well as disruption of the molecular networks in the CNS. In this current study, we investigated whether HIV-1-infected MDMs have any role in causing Ng loss. Using HIV-1-infected MDM supernatants, we have shown that Ng was majorly abrogated from the cytoplasm of dSH-SY5Y cells, whereas the nuclear concentration of Ng did not show significant change. Whether this loss of Ng from the dendrites/cytoplasm is due to direct effect of the virus or an outcome of increased inflammation caused by viral infection is not well established.

The role of HIV-1 viral and cellular proteins in neuronal injury has been documented in multiple studies. Among the viral proteins, gp120 and Tat are more common to exert a functional role in disrupting neuronal plasticity [48]. HIV-1 Tat overexpresses histone deactylase 2 (HDAC2) in neuronal cells that results in downregulation of CaMKII and CREB leading to impaired synaptic plasticity [49]. On the other hand, release of these viral proteins disrupts the BBB leading to infiltration of more HIV-1-infected monocytes into the CNS. These infected cells in turn produce more viral proteins, inflammatory cytokines, and neurotoxins, which may be the major factors causing disruption of neuronal damage. HIV-1-associated inflammation has marked effects on HAND pathology. Neuroinflammation makes the dendrites and synapses more susceptible to excitotoxic injury [50]. The proinflammatory cytokines/chemokines produced excessively in the brain during chronic inflammation including tumor necrosis factor (TNF)-α, IL-1β, and IL-8 have profound effects on synaptic transmission and plasticity [51, 52]. Elevated levels of TNF-α during chronic inflammation modulate synaptic signaling through activation of TNFR1 receptors on pre- and post-synaptic neurons [53–55]. Elevated levels of IL-1β and IL-8 secreted by brain macrophages in the HIVE patients inhibit the induction of LTP [56, 57]. We focused on the roles of host inflammatory factors, because studies have shown that viral toxicity is induced by inflammatory host-derived cofactors [58] and also viral proteins such as gp120 causes neuronal damage through upregulating inflammatory cytokines [22]. We have shown for the first time that in HIV-1-infected subjects, inflammatory IL-1β and IL-8 levels are associated with Ng downregulation and synaptodendritic injury. In contrast to other studies, TNF-α level was not significantly (*p* = 0.0625) elevated in HIV-1-infected MDMs compared to controls, although similar trend was observed. One possible explanation could be the difference in time of collection of MDM supernatants after infection or MOI used for infection. Treatment of dSH-SY5Y cells with HIV-1-infected MDM supernatants markedly reduced Ng as well as CaMKII and synaptic markers Syp and Syn I. Treatment with recombinant proteins and neutralizing antibodies further confirmed our results. Together, chronic HIV-1 infection alters the inflammatory milieu in the CNS compartment leading to downregulation of Ng, which is associated with at least in part with the synaptodendritic injury.

## Conclusion

This is the first study evaluating the expression and functional role of Ng in the context of HIV-1 neuropathogenesis. Here, we demonstrate that HIV-1 infection decreases the level of Ng in human FC tissues in HAD patients and this decrease is in part due to overexpression of proinflammatory cytokines IL-1β and IL-8. The reduced expression of Ng in HAD subjects results in a limited interaction with CaM in the dendritic spines that dysregulates the downstream CaM-CaMKII signaling pathway. This in turn abrogates the synaptic plasticity measured by the reduced expression of Syp and Syn I. These results demonstrate one of the mechanisms of cognitive impairment in HAD-positive subjects through Ng dysregulation.
